# Tick Control Strategies: Critical Insights into Chemical, Biological, Physical, and Integrated Approaches for Effective Hard Tick Management

**DOI:** 10.3390/vetsci12020114

**Published:** 2025-02-02

**Authors:** Tsireledzo Goodwill Makwarela, Nimmi Seoraj-Pillai, Tshifhiwa Constance Nangammbi

**Affiliations:** Department of Nature Conservation, Faculty of Science, Tshwane University of Technology, Staatsartillerie Rd, Pretoria West, Pretoria 0183, South Africa; seorajpillayn@tut.ac.za (N.S.-P.); nangammbitc@tut.ac.za (T.C.N.)

**Keywords:** acaricides, biological control, hard ticks, integrated pest management, sustainable management, tick control, wildlife–livestock interface

## Abstract

Ticks and tick-borne diseases significantly impact animal health, public health, and economic productivity globally, particularly in areas where the wildlife–livestock interface complicates management. This review critically examines the current control strategies, focusing on chemical, biological, physical, and integrated pest management (IPM) approaches. Chemical acaricides, while effective, are increasingly challenged by resistance development and environmental concerns. Biological approaches, including natural predators and entomopathogenic fungi, and physical interventions, such as habitat modification, provide sustainable alternatives but require further optimization. IPM stands out as the most promising long-term solution, integrating multiple approaches to enhance efficacy while reducing environmental risks. Emerging innovations, such as nanotechnology-enhanced acaricides and next-generation vaccines, offer promising avenues for improved tick control. Addressing the complex challenges of tick management requires tailored strategies, interdisciplinary collaboration, and sustained research investment in both veterinary and public health contexts.

## Simple Summary

Ticks and tick-borne diseases (TBDs) are a global concern, impacting public health, veterinary care, and economic productivity. This review evaluates the effectiveness of various control strategies, highlighting the limitations of chemical acaricides due to resistance and environmental impact. Alternative methods, including biological controls like entomopathogenic fungi and natural predators, show promise but face scalability challenges. Physical strategies, such as habitat management, complement these approaches, while emerging innovations, including microbiota-targeted techniques and next-generation vaccines, offer new opportunities for sustainable tick control. The findings emphasize the need for affordable, integrated solutions tailored to specific contexts and supported by interdisciplinary collaboration under a One Health framework.

## 1. Introduction

### 1.1. Ticks and Their Importance as Vectors of Human and Animal Diseases

**Figure 1:**
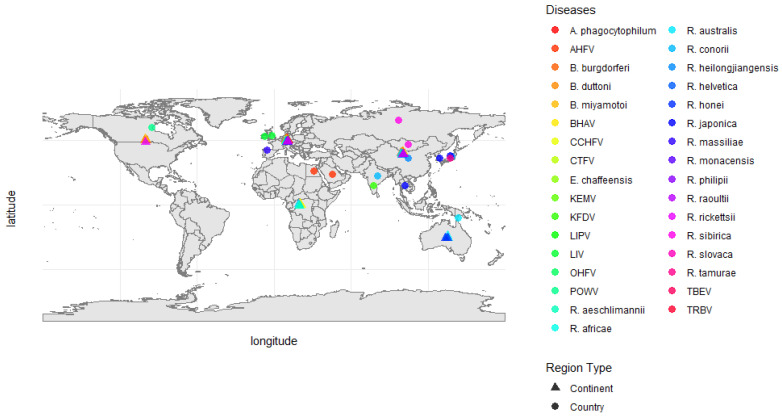
Global distribution of medically important tick-borne viruses and bacteria. This map illustrates the geographic distribution of tick-borne diseases, with different colors representing specific pathogens, including viral diseases such as tick-borne encephalitis virus (TBEV), Crimean–Congo hemorrhagic fever virus (CCHFV), Colorado Tick Fever Virus (CTFV), Powassan Virus (POWV), and Kyasanur Forest Disease Virus (KFDV), as well as bacterial species such as *Borrelia burgdorferi* (Lyme disease), *Anaplasma phagocytophilum*, *Ehrlichia chaffeensis*, and various *Rickettsia* species. The shapes in the map differentiate the level of geographic reporting, where triangles (▲) represent data aggregated at the continental level, and circles (●) indicate data at the country level, with overlapping points slightly offset to enhance readability and avoid visual congestion in high-density regions. The data presented in this map were compiled from multiple surveillance studies, which were summarized in a table in a study by Vilibić-Čavlek [8].

Ticks are significant vectors of various pathogens, including bacteria, viruses, and protozoa, which cause diseases such as Lyme disease, anaplasmosis, and tick-borne encephalitis (TBE) [1]. These diseases have varying geographic distributions and impacts globally, as illustrated in Figure 1, highlighting the spread of tick-borne human diseases across different continents and countries. The diverse health impacts of ticks on humans and animals are summarized in Table 1, highlighting their role in disease transmission, economic losses, and public health risks. The rising incidence of these diseases has been attributed to factors such as environmental changes, urbanization, and climate change, which have contributed to the expanded geographic range and increased population density of ticks [2,3]. For example, the black-legged tick (*Ixodes scapularis*), a primary vector of Lyme disease, has expanded its geographic range significantly, raising concerns about human exposure to Lyme disease and other tick-borne illnesses [4]. Historically concentrated in the northeastern and upper midwestern United States, *I. scapularis* is now increasingly reported in southern Canada and southeastern U.S. regions previously considered non-endemic [5]. Addressing these challenges requires a comprehensive understanding of tick ecology and the factors influencing their distribution, which can inform the development of effective public health interventions [6,7].

Ticks pose a substantial threat to livestock health, causing conditions such as anemia, weight loss, and even mortality in cases of severe infestations [17,18,19,20]. Economically, tick infestations are highly detrimental, with losses from tick-borne diseases in cattle alone estimated to reach hundreds of millions of dollars annually [21,22]. Effective management strategies, including the use of acaricides, and vaccines are critical for safeguarding livestock health and productivity [23]. However, the increasing development of resistance to chemical treatments highlights the need for integrated pest management (IPM) approaches that combine multiple strategies to achieve sustainable tick control [24]. Public health initiatives frequently focus on education and awareness campaigns to encourage preventive behaviors among populations at risk of tick exposure. Studies demonstrate that improving knowledge about tick-borne diseases and preventive measures leads to increased protective behaviors, such as wearing appropriate clothing, using repellents, and conducting thorough tick checks after outdoor activities [25,26]. In areas with high tick populations, public health messaging emphasizes avoiding tick habitats and performing regular tick checks, particularly during peak seasons [27,28,29]. These educational efforts are essential for reducing the frequency of tick bites and the subsequent transmission of pathogens.

**Figure 2:**
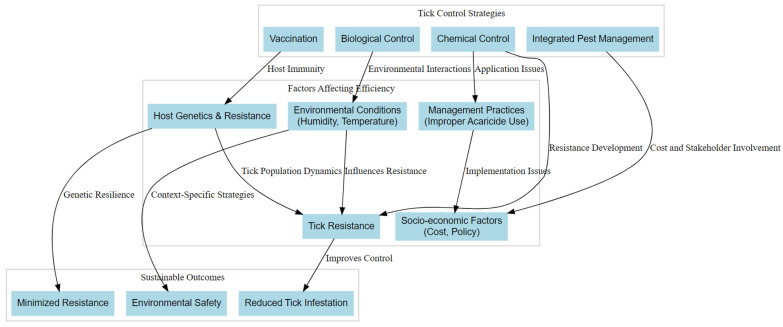
The interaction between tick control strategies and factors affecting their efficiency. It highlights the central role of chemical, biological, and integrated control methods, their interdependence with resistance, host genetics, environmental conditions, and socio-economic factors, with a focus on sustainable outcomes. The data presented in this chart were compiled from [36,37,38,39,40].

Surveillance plays a critical role in tick management by providing essential data on tick populations and their pathogens. Passive surveillance methods, such as collecting ticks from the environment or public submissions, offer valuable insights into tick distribution and pathogen prevalence [30,31]. Such data inform public health responses and guide targeted interventions in high-risk areas [6]. Moreover, integrating advanced technologies, such as geographic information systems (GISs) and molecular techniques, enhances the accuracy and efficiency of surveillance efforts [32,33]. The One Health approach, which recognizes the interconnectedness of human, animal, and environmental health, promotes collaboration among public health officials, veterinarians, and environmental scientists to address the complex challenges posed by ticks and tick-borne diseases [34]. By fostering interdisciplinary partnerships, stakeholders can develop comprehensive strategies that not only control tick populations but also address broader ecological factors influencing disease transmission [35]. Effective tick control strategies require an integrated approach that combines chemical, biological, and managerial methods to address the multi-faceted challenges of acaricide resistance, environmental conditions, and socio-economic factors. A conceptual framework illustrating these interactions is shown in Figure 2.

### 1.2. The Economic Impact of Ticks and Tick-Borne Diseases

#### 1.2.1. Direct Economic Losses from Ticks and TBDs

Ticks and their associated diseases cause direct financial losses, particularly in livestock production. For instance, the economic impact of ticks and TBDs globally is estimated to reach as much as USD 22–30 billion/annum with the largest share attributed to livestock mortality and morbidity, especially in cattle and small ruminants [41]. Tick infestations result in blood loss, anemia, reduced weight gain, and decreased milk production, all of which affect farmers’ profitability [42,43,44]. Additionally, managing tick populations and treating infected animals incur significant costs, further straining livestock producers’ resources [45].

#### 1.2.2. Indirect Economic Costs

Beyond direct impacts, the indirect costs of TBDs are equally significant. The treatment of human diseases such as Lyme disease and tick-borne encephalitis imposes a heavy financial burden on healthcare systems. In the United States alone, annual Lyme disease treatment costs are estimated to be between USD 345 and 968 million, driven by the disease’s rising incidence [46]. Furthermore, economic downturns often exacerbate the incidence of TBDs by reducing funding for public health initiatives, leading to less effective disease management and prevention strategies [47]. The economic implications of TBDs are particularly severe in developing countries, where livestock farming is central to livelihoods and food security. In sub-Saharan Africa, for example, losses attributed to TBDs significantly hinder agricultural productivity [48,49,50]. Diseases like tropical theileriosis and anaplasmosis have high mortality rates in cattle, leading to catastrophic financial losses for local farmers [51,52,53,54]. Veterinary expenses, including vaccinations and treatments, further compound these challenges, making it difficult for resource-limited farmers to sustain their operations [55]. Acaricide resistance has become a critical issue, particularly in Ecuador’s dairy farms, where the cattle tick *Rhipicephalus microplus* exhibits high resistance levels to amitraz, ivermectin, and, a recent alternative, alpha-cypermethrin in about 42%, 39%, and 24% of farms, respectively [56]. Similarly, a study in Brazil demonstrated that chemical treatments for *R. microplus* impose a heavy financial strain on small-scale farmers, exacerbated by resistance, which forces more frequent or alternative treatments [57]. Climate change and habitat modification have further amplified the challenges associated with ticks and TBDs. As ticks adapt to changing environments, their geographic range expands, increasing the incidence of TBDs in areas previously considered low-risk [2,3,58]. This expansion impacts livestock and human populations, driving up healthcare costs and productivity losses.

### 1.3. Tick Life Cycles

Ticks, classified under the order *Ixodida*, are ectoparasitic arachnids that play a significant role in transmitting various pathogens affecting both human and animal health. This order is divided into three primary families: *Ixodidae* (hard ticks), *Argasidae* (soft ticks), and *Nuttalliellidae*. Among these, the *Ixodidae* family is particularly noteworthy due to its extensive involvement in vectoring diseases such as Lyme disease, anaplasmosis, and babesiosis, caused by various pathogens, including bacteria and protozoa [59,60]. The *Ixodidae* family is characterized by a hard-bodied structure featuring a scutum, a shield-like feature that partially covers the dorsal side of the tick [61]. Hard ticks typically attach to their hosts for extended periods, enabling them to feed more extensively and thereby increasing their potential to transmit pathogens [58]. In terms of species diversity, the *Ixodidae* family comprises approximately 700 species, while the *Argasidae* family contains around 170 species. The *Nuttalliellidae* family, in contrast, is represented by a single species, *Nuttalliella Namaqua* [62].

These ectoparasitic arachnids have complex life cycles, typically characterized by three developmental stages, larva, nymph, and adult, with each stage requiring a blood meal for development and presenting distinct host preferences and pathogen transmission risks [63]. The number of hosts utilized significantly influences their capacity for disease transmission [64]. Understanding these distinctions is crucial for elucidating the epidemiology of tick-borne diseases and developing effective control strategies. The life cycle of *Ornithodoros* ticks is complex and varies by species, which is crucial for designing targeted control strategies [65]. For example, *Ornithodoros moubata*, a key vector of African swine fever virus (ASFV), progresses through distinct life stages, larva, nymph, and adult, each with specific feeding behaviors and ecological requirements [66].

#### 1.3.1. Hard Ticks (Ixodidae)

Hard ticks are obligate hematophagous ectoparasites that require blood meals from their hosts to complete their life cycle, which includes the larval, nymphal, and adult stages [67]. These ticks are known for their prolonged feeding behavior, which can last several days. During this period, they can transmit various pathogens, including bacteria, viruses, and protozoa [58,68]. Prominent diseases transmitted by hard ticks include Lyme disease, caused by *B. burgdorferi*, and tick-borne encephalitis, among others [69]. The evolutionary history of hard ticks suggests they diverged from soft ticks approximately 120 to 92 million years ago, a timeline reflected in their distinct physiological and behavioral adaptations [70]. Hard ticks possess specialized mouthparts that anchor securely to their hosts during feeding, while their saliva contains anticoagulants that prevent blood clotting and modulate the host’s immune response [71,72]. This saliva also contains a variety of proteins that facilitate feeding by suppressing host immune defenses [71]. Research has demonstrated that hard ticks exhibit a high degree of host specificity, although they can infest a wide range of vertebrate hosts, including mammals, birds, and reptiles [73,74]. This adaptability is essential for their survival and reproduction, as the availability of suitable hosts significantly influences their population dynamics [2,75]. Additionally, environmental factors such as temperature, humidity, and vegetation play crucial roles in determining the distribution and abundance of hard tick populations [62,76,77,78,79].

#### 1.3.2. Soft Ticks (Argasidae)

In contrast, soft ticks from the *Argasidae* family exhibit markedly different feeding behaviors and life cycles. Typically, they are nocturnal feeders, and they take shorter and more frequent blood meals compared to hard ticks [80]. Soft ticks possess a flexible body structure, enabling them to hide in crevices and remain undetected by their hosts [81]. Soft ticks are known to transmit several pathogens, including those responsible for tick-borne relapsing fever, which is primarily caused by *Borrelia* species [10,82]. Unlike hard ticks, soft ticks do not attach firmly to their hosts; instead, they feed quickly and retreat to hiding places, making detection and control more challenging [83]. The presence of multiple host species facilitates pathogen maintenance and transmission within tick populations, making it difficult to disrupt the infection cycle. *I. ricinus* relies on a range of hosts, from small mammals like the white-footed mouse (a key reservoir for *B. burgdorferi*, the pathogen causing Lyme disease) to larger mammals such as deer, which are essential for adult tick reproduction [84,85]. Similarly, *R. microplus* primarily infests cattle, serving as a vector for *Babesia bovis* and *Anaplasma marginale*, while *Amblyomma americanum* targets diverse hosts, transmitting *E. chaffeensis* to humans and livestock [86]. More examples are illustrated in Table 2.

#### 1.3.3. Host Utilization Strategies

Ticks display a variety of life cycle strategies, categorized as one-host, two-host, and three-host cycles, each with specific ecological and epidemiological implications. In one-host ticks, such as *R. microplus* and *R. annulatus*, all parasitic stages, including the larva, nymph, and adult, develop on a single host [75,88]. The ticks detach only to lay eggs, which minimizes host-seeking effort and conserves energy. However, this dependency on a single host increases vulnerability to host availability. One-host ticks like *R. microplu* are known vectors of several pathogens, including *A. marginale*, *Anaplasma centrale*, *Babesia bigemina*, and *B. bovis* [89,90].

Two-host ticks, including *Hyalomma anatolicum* and *H. marginatum*, use one host for their larval and nymphal stages. After engorgement, the nymph detaches, molts into an adult, and seeks a second host for feeding and reproduction [91]. This strategy allows ticks to exploit a broader range of hosts, enhancing their adaptability. For instance, *H. marginatum* is a well-known vector of the Crimean–Congo hemorrhagic fever virus, a significant zoonotic pathogen [87,89].

Three-host ticks, such as *I. ricinus*, feed on various animals such as small mammals, livestock, birds, reptiles, and humans [92]. This life cycle increases exposure to a wide variety of host species, making these ticks highly effective vectors for multiple pathogens. For example, *Ixodes scapularis* transmits Lyme disease and babesiosis, while *A. hebraeum* is a vector of heartwater disease caused by *E. ruminantium* [93,94]. Although this life cycle provides ecological flexibility, it requires greater energy investment due to repeated host-seeking [89].

Soft ticks, from the family Argasidae, such as *O. moubata*, exhibit a multi-host life cycle where multiple blood meals are required during the nymphal stages [95]. Unlike hard ticks, soft ticks feed rapidly, often completing a meal within an hour and detaching promptly [96]. These ticks are key vectors of relapsing fever caused by *Borrelia* species [97]. Their life cycle is adapted to sheltered environments such as animal burrows or nests, enabling repeated feedings on hosts nearby [89].

### 1.4. Host Specificity and Implications for Pathogen Transmission

Ticks’ host specificity and feeding behavior significantly influence disease transmission dynamics. For example, a decline in competent reservoir hosts can reduce pathogen transmission, potentially leading to the localized extirpation of both the tick and the pathogen [68,98,99,100,101]. Climate change and habitat fragmentation also alter host availability and tick life cycle timing, increasing interactions between ticks and hosts [102]. Warmer temperatures can extend the questing season, providing more opportunities for feeding and pathogen transmission [103].

**Figure 3:**
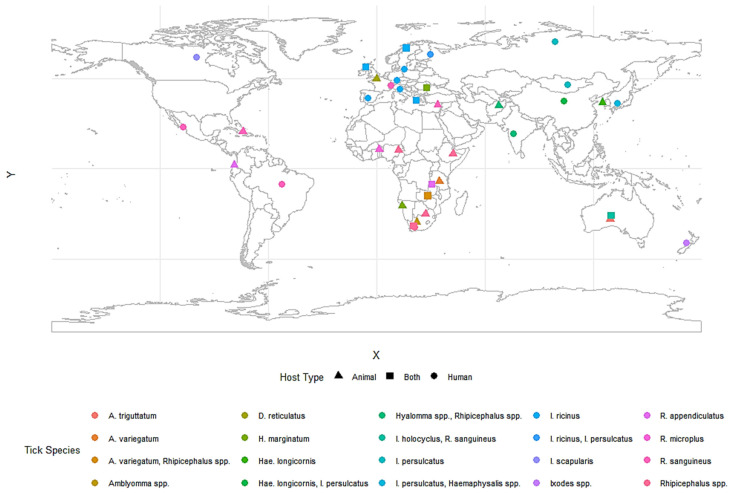
Global distribution of common tick species by host type. This map shows the global distribution of common tick species and their association with host types, based on verified research and surveillance data. Host types are shown as shapes: circles (●) for humans, triangles (▲) for animals, and squares (■) for both. Tick species are distinguished by colors, as shown in the legend using data from regional studies, including [13,35,109,110,111,112,113,114,115,116,117,118,119,120,121,122,123,124,125,126,127,128,129,130,131,132,133,134,135,136,137,138,139,140,141,142].

The adaptability of ticks to diverse environments and their reliance on multiple hosts complicates management strategies. In Africa, species like *R. microplus* and *A. variegatum* in South Africa and Nigeria primarily target livestock, causing diseases such as anaplasmosis and babesiosis [104,105]. In Asia, *Haemaphysalis longicornis* in China and South Korea parasitizes livestock and serves as a vector for Lyme disease and severe fever with thrombocytopenia syndrome [9]. In Europe, countries like Germany and Italy experience mixed host interactions involving *Ixodes ricinus*, which parasitizes both livestock and humans, contributing to Lyme disease and tick-borne encephalitis [106]. Urban-adapted ticks, such as *R. sanguineus*, thrive in urban settings, feeding primarily on dogs and occasionally humans. This behavior facilitates the spread of pathogens like *Ehrlichia* and *Rickettsia*, particularly in densely populated areas [107,108]. These patterns of tick–host interactions are further illustrated in Figure 3, which maps the global distribution of common tick species and their associations with host types.

Ticks are not only vectors but also reservoirs for a wide array of pathogens, including bacteria, viruses, and protozoa, which cause severe diseases in humans and animals [10,74,143,144]. For example, North America is dominated by *I. scapularis* and *Dermacentor variabilis* in the United States, which are primary vectors for Lyme disease, anaplasmosis, and babesiosis [145]. In Canada, *I. scapularis* and *Ixodes pacificus* contribute to similar disease burdens [146]. Contrastingly, regions in South America, such as Brazil and Argentina, are heavily affected by *R. microplus*, a major vector of bovine babesiosis and anaplasmosis [147,148,149,150]. In Oceania, species such as *Ixodes holocyclus* in Australia and New Zealand are responsible for tick paralysis and emerging Lyme-like diseases [6].

This review aims to critically evaluate the current strategies for controlling hard tick populations and mitigating the transmission of tick-borne diseases. By examining chemical, biological, and integrated approaches, this study seeks to identify effective, sustainable, and ecologically sound methods to address the growing challenges posed by ticks in both public health and veterinary contexts. Particular emphasis is placed on understanding the limitations of conventional acaricides, exploring innovative biological controls, and assessing the potential of integrated pest management systems. Ultimately, the insights provided aim to inform future research, guide policy development, and promote holistic approaches to hard tick control, ensuring the sustainability of interventions and the health of ecosystems.

## 2. Chemical Control

Historically, chemical acaricides such as organophosphates and pyrethroids have served as the primary tools for tick control due to their effectiveness in reducing infestations and preventing tick-borne diseases [6,151]. However, the extensive use of these chemicals led to widespread resistance, diminishing their efficacy and prompting the search for alternative strategies [23,152,153]. Acaricides are classified based on their modes of action, which range from neurotoxins, such as organophosphates, pyrethroids, and phenylpyrazoles, to growth regulators and chitin synthesis inhibitors [154]. The diversity of acaricide classes, their examples, and their respective modes of action are summarized in Table 3. This overview highlights the distinct mechanisms by which acaricides target tick populations, providing insights into their applications and challenges associated with resistance.

### 2.1. Methods of Acaricide Application

One common method for applying acaricides is dipping, where livestock are submerged in a solution containing the acaricide [55]. This ensures comprehensive coverage, including hard-to-reach areas, and is widely used in regions like South Africa for controlling tick populations [161,162]. However, factors such as acaricide concentration, exposure duration, and organic matter in the solution can compromise effectiveness. Plunge dipping requires careful management to prevent resistance, which is increasingly reported globally [163,164]. Spraying, often used when dipping is impractical, allows for the targeted treatment of individual animals using handheld or motorized sprayers. While effective, uneven coverage in densely packed animals and improper acaricide dilution can reduce efficacy and promote resistance [165,166,167].

Pour-on formulations offer a labor-efficient alternative, applying the acaricide directly onto the animal’s back for slow release over time. This method’s ease of use makes it popular, but coverage issues, particularly concerning wet or dirty coats, and resistance concerns remain challenges [168]. Alternative approaches, such as biopesticides and plant-derived products, are gaining attention as sustainable options. These can be applied similarly to traditional methods and may lower the risk of resistance [169,170,171]. Environmental and economic factors influence the choice of application method. Resource-limited regions may favor less expensive but less effective methods [171]. Tick-borne diseases often drive frequent applications to mitigate livestock losses, but indiscriminate use risks environmental contamination and chemical residues in meat and milk [62].

### 2.2. Repellents

#### 2.2.1. DEET (N,N-Diethyl-meta-toluamide)

DEET is one of the oldest and most extensively studied insect repellents, having been introduced in the 1950s [172]. Its effectiveness against various tick species, including *A. americanum* and *I. scapularis*, has been well documented. Studies indicate that DEET provides significant protection, with formulations containing 20% DEET offering up to 12 h of protection against tick bites [173]. However, the efficacy of DEET can vary based on concentration and environmental conditions. For instance, some research suggests that lower concentrations may not provide adequate protection, particularly against certain tick species [174]. Despite its widespread use, DEET is not without concerns. Reports have indicated potential neurotoxic effects at high concentrations, raising questions about its safety, particularly for vulnerable populations such as children [175].

#### 2.2.2. Picaridin

Picaridin, developed as a synthetic alternative to DEET, has gained popularity due to its favorable safety profile and effectiveness [176]. Research has shown that Picaridin is comparable to DEET in terms of efficacy against ticks, with some studies suggesting that it may even outperform DEET in certain contexts [177]. Picaridin is less likely to cause skin irritation and has a more pleasant odor, making it a preferable option for many users [178]. Field studies have demonstrated that Picaridin can provide long-lasting protection, often exceeding that of DEET under similar conditions [179,180]. Its mechanism of action involves disrupting the sensory receptors of ticks, thereby preventing them from locating hosts effectively [181]. This makes Picaridin a valuable option for individuals seeking effective tick protection without the drawbacks associated with DEET.

#### 2.2.3. IR3535 (Ethyl Butylacetylaminopropionate)

IR3535 is another synthetic repellent that is effective against ticks. It operates through a similar mechanism to DEET and Picaridin, interfering with the sensory perception of ticks [182,183,184]. Research indicates that IR3535 can provide substantial protection, although it may not be as widely recognized or utilized as DEET or Picaridin [185,186].

#### 2.2.4. Botanical Repellents

The appeal of botanical repellents lies in their perceived safety and environmental friendliness. However, their efficacy can be inconsistent, often influenced by factors such as concentration and formulation [187,188]. Additionally, while some botanical repellents may offer short-term protection, they often require more frequent application compared to synthetic options like DEET and Picaridin [189]. Natural repellents derived from essential oils and plant extracts provide eco-friendly alternatives to synthetic chemicals [190,191]. Table 4 highlights a variety of natural repellents, along with their botanical sources, active compounds, and efficacy against various tick species. For example, lemongrass (*Cymbopogon citratus*) oil, containing carvacrol, has shown high mortality rates against *R. microplus* in laboratory studies [192]. Eucalyptus (*Eucalyptus* spp.) and thyme (*Thymus vulgaris*) oils, with active compounds like thymol and eugenol, have demonstrated strong acaricidal and repellent effects against *I. ricinus* [193,194]. Plant extracts, including those from tobacco (*Nicotiana tabacum*) and eucalyptus (*Eucalyptus globoidea*), have effectively deterred tick attachment and feeding [187,195,196]. Nootkatone, a sesquiterpene found in grapefruit and cedar, offers comparable efficacy to DEET against *I. scapularis* and *A. americanum*, making it a compelling natural option for large-scale use [197].

### 2.3. Environmental Chemical Treatments

Environmental chemical treatments, including pesticides and soil treatments, play a crucial role in managing tick populations by targeting their off-host environments, such as vegetation, soil, and buildings. Pesticides, particularly acaricides, effectively reduce tick populations in treated areas, with optimized spraying techniques enhancing their efficiency [210]. For instance, permethrin-treated materials have been shown to effectively lower *I. scapularis* populations, thereby reducing Lyme disease risks in small mammals [211]. Long-lasting permethrin-impregnated (LLPI) clothing has been demonstrated to retain bioactive levels of permethrin and achieve high tick mortality rates, with up to 88% effectiveness after three months of real-world use, making it a practical intervention for tick bite prevention [212]. A comparative study on the efficacy of chlorpyriphos and deltamethrin against bovine ticks demonstrated that chlorpyriphos exhibited a prolonged residual effect, with reinfestation observed in 28.57% of treated animals after 14 days, whereas deltamethrin-treated animals experienced a faster reinfestation rate, with 50% showing tick presence within the same period [213].

### 2.4. Challenges to Chemical Control

#### 2.4.1. Resistance

**Figure 4:**
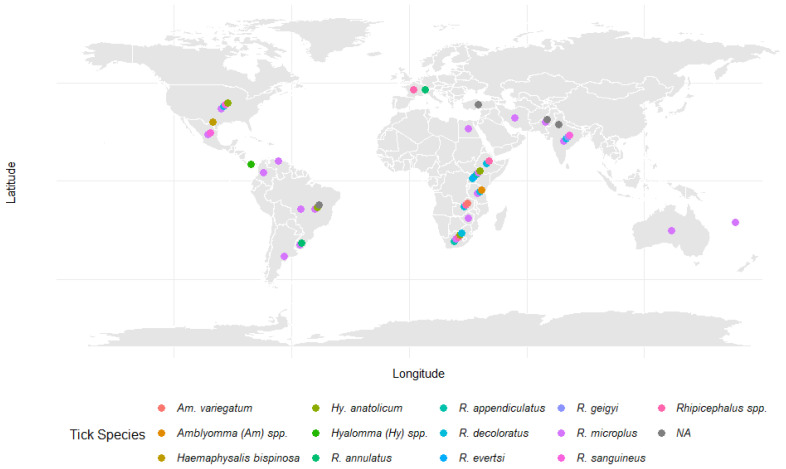
Global distribution of tick species resistant to common control methods. This map illustrates the global distribution of tick species exhibiting resistance to conventional control measures. Each colored point represents a unique tick species identified in a specific country. Points within a country are spread to visually distinguish multiple resistant species. Data were compiled from various studies, as detailed in the accompanying table, with significant contributions from Obaid [23].

Synthetic acaricides, such as spirodiclofen and fenazaquin, have demonstrated high efficacy in controlling agricultural pests and ticks. For example, a combination of 10% imidacloprid and 50% permethrin (Advantix, Bayer Animal Health) achieved a tick efficacy of up to 97.9% against *R. sanguineus* (s.l.) when applied every 21 ± 2 days over a year, with post-treatment efficacy ranging between 96.1% and 98.9% for 4 weeks following spot-on administration revision [214]. However, some synthetic acaricides, like clofentezine, showed variable performance, emphasizing the importance of selecting appropriate compounds for specific applications [215]. Natural acaricides derived from plant extracts are gaining attention for their reduced environmental impact. Essential oils from plants like *Tagetes minuta*, *Syzygium aromaticum*, and *C. citratus* have shown significant acaricidal properties against ticks [152,216]. Although these botanical alternatives can be effective, their performance often depends on concentration, active compound composition, and formulation. The addition of surfactants has been shown to enhance the efficacy of aqueous plant extracts, making these botanical alternatives more effective [217]. Resistance development remains a critical challenge in acaricide use. The continuous and indiscriminate application of acaricides has led to the widespread development of resistance in tick populations, particularly in economically important species such as *Rhipicephalus (Boophilus) microplus* and *R. sanguineus*, with resistance reported against various acaricide classes, including pyrethroids, organophosphates, and amidines across multiple countries and regions [163]. Resistance mechanisms include genetic mutations altering target sites, enhanced metabolic detoxification, and behavioral adaptations [218,219]. For instance, the overexpression of ATP-binding cassette (ABC) transporters in ticks has been linked to increased resistance, significantly reducing acaricide efficacy [220,221,222]. Resistance to acaricides is a significant global challenge in tick control, as illustrated in Figure 4 and detailed in Table 5. Despite their widespread use as the primary method for managing tick populations, multiple economically important tick species have developed resistance to commonly used acaricides, including pyrethroids, organophosphates, and macrocyclic lactones.

#### 2.4.2. Environmental Toxicity

Chemical acaricides are effective in controlling ticks but pose significant environmental and health risks, emphasizing the need for sustainable alternatives. Environmental contamination is a major concern, as many acaricides persist in soil and water, indiscriminately harming non-target agents like pollinators as well as aquatic life [240]. Runoff from treated areas can lead to bioaccumulation in aquatic ecosystems, disrupting biodiversity and potentially entering the human food chain [241]. Environmental factors, including climate, habitat characteristics, and human activity, play a critical role in shaping tick populations and influencing the success of control measures [242]. Climate change has emerged as one of the most significant drivers of tick population dynamics, altering their geographic distribution and activity levels. Changes in temperature and precipitation patterns allow ticks to expand into previously inhospitable regions, increasing the range of species such as *I. ricinus* and raising the risk of Lyme disease transmission in new areas [2,3,243]. For example, research in the United Kingdom and North America demonstrates that warmer temperatures and higher humidity levels enhance tick survival and activity, necessitating adaptive management strategies [244]. Seasonal variability further complicates tick control, as ticks exhibit heightened activity during warmer months, leading to increased encounters with humans and animals [242]. Habitat characteristics, including vegetation type and land use, also significantly influence tick populations. Dense vegetation, such as forests with tall grass and leaf litter, provides the humidity and shelter necessary for tick survival and questing behavior [245]. Conversely, habitat modifications, such as the removal of leaf litter and vegetation, can dramatically reduce tick populations [246]. However, urbanization and land-use changes often create fragmented habitats that serve as new niches for ticks, increasing human–tick interactions in urban and peri-urban environments [247,248].

#### 2.4.3. Non-Target Impacts

Notably, declines in pollinator populations, such as honey bees, have also been linked to acaricide use, threatening agricultural productivity and ecological balance [249,250]. The health risks of acaricides additionally extend to agricultural workers and consumers. Acute and chronic issues, including neurotoxicity from organophosphates, are well documented, with children and pregnant women being particularly vulnerable [251,252,253]. Residues in food products like meat and eggs raise concerns about dietary exposure, which has been associated with endocrine disruption and increased cancer risks [254,255,256].

#### 2.4.4. Impact of Environment on the Efficacy of Chemicals

High temperatures increase evaporation rates, reducing the effective concentration of certain compounds and diminishing their acaricidal activity [257]. Conversely, lower temperatures can slow the metabolic processes of pests, allowing them to survive doses that would otherwise be lethal under optimal conditions [258]. Global warming further accelerates pesticide degradation, reducing environmental persistence and long-term efficacy [259]. Humidity levels also impact acaricide performance. While high humidity enhances the retention and absorption of liquid formulations, improving efficacy, excessive moisture can degrade some chemical compounds, reducing their residual activity. For instance, acaricides such as deltamethrin and alphacypermethrin show varying efficacy under different humidity conditions, emphasizing the importance of environmental monitoring during application [260]. Low humidity, on the other hand, can lead to rapid evaporation and increased pesticide drift, as highlighted by studies by Silva [261] and Stanislavski [262]. Surface-applied treatments can be washed away by rain, significantly reducing their effectiveness and necessitating reapplication [263]. Soil type also plays a role, with sandy soils promoting leaching and clay soils prolonging acaricide retention and effectiveness [264].

These variables also affect the soil–air partitioning of semivolatile pesticides, influencing their availability and persistence [265,266]. Beyond direct effects on acaricide performance, environmental factors also influence pest behavior and susceptibility. Warmer temperatures, for instance, may alter pest tolerance to acaricides, accelerating resistance development [267]. Wind speed and humidity can constrain the timing of applications, requiring precise planning to maximize efficacy [268].

## 3. Biological Control

### 3.1. Biological Control Agents for Tick Management

Recent research on *Lasius alienus* has demonstrated its predation on tick eggs, with findings indicating that the presence of egg wax significantly influences predation rates [269]. Birds like oxpeckers (*Buphagus* spp.) also contribute to tick removal from large mammals, although their effectiveness depends on host availability and environmental conditions [270]. Parasitoid wasps, particularly *Ixodiphagus hookeri*, parasitize ticks by laying eggs inside them, with larvae feeding on and ultimately killing the ticks, especially *A. variegatum* populations [271].

Entomopathogenic fungi, such as *Metarhizium anisopliae* and *Beauveria bassiana*, present another promising biological control option. These fungi infect ticks through spore germination and cuticle penetration, causing fatal mycosis [272]. Studies have demonstrated significant reductions in tick populations following fungal treatment. For instance, the application of *M. anisopliae* to residential areas resulted in a 55.6% reduction in nymphal tick abundance on lawns and an 84.6% reduction in woodland plots [273]. Their effectiveness depends on environmental factors, particularly temperature and humidity, which influence fungal virulence and persistence. High temperatures, low humidity, and UV exposure can reduce efficacy by impairing spore germination and survival [274]. Despite these challenges, *M. anisopliae* and *B. bassiana* are valuable for integrated pest management (IPM) due to their host specificity and low resistance risk. However, regional environmental conditions must be considered for optimal application and effectiveness [275].

### 3.2. Challenges and Considerations

The effectiveness of entomopathogenic fungi (*M. anisopliae* and *B. bassiana*) in tick control is challenged by reduced field efficacy, strain selection, and optimization of application methods and timing [276]. Furthermore, the persistence and effectiveness of fungal spores in natural environments can be reduced by exposure to ultraviolet radiation, necessitating optimized application strategies and timing for maximum impact [277]. The use of entomopathogenic fungi (EPFs) for biological tick control faces several challenges. Delayed mortality is a major issue, as EPFs take days to kill ticks, allowing them to transmit *T. parva* before dying. This slow action provides only indirect protection at the population level rather than immediate relief [278]. Another limitation is the short persistence of EPFs on cattle skin, often lasting only one to three days. This requires frequent reapplications, adding to labor and cost burdens [279]. Additionally, EPFs may prolong tick engorgement, increasing the risk of pathogen transmission before the tick dies [280].

## 4. Physical Control

### Habitat Management Strategies and Natural Tick Repellents

Habitat management reduces tick populations by modifying environmental conditions to make them less favorable. Techniques such as forest thinning and canopy reduction create drier microclimates, lowering tick survival by reducing humidity [281]. Prescribed burning, which eliminates organic material where ticks reside, has shown short-term effectiveness in disrupting tick life cycles, though long-term results vary by ecosystem [282]. Host management strategies, such as controlling deer densities through culling or fencing, have demonstrated significant reductions in tick populations and Lyme disease risks in studies conducted in the southeastern United States and Scotland [283]. Ecological restoration, including the promotion of native vegetation and natural predators like tick-feeding birds, further enhances sustainable tick control [284,285].

## 5. Mechanical Control

### 5.1. Manual Removal, Tick Traps, and Host Grooming

Manual removal using tools like tick twisters or fine-tipped tweezers minimizes damage to tick mouthparts, thereby reducing pathogen transmission risks [286]. Techniques such as twisting or lassoing are particularly effective for species like *I. ricinus* and *Dermacentor reticulatus*, which transmit Lyme disease and babesiosis, respectively [287]. In resource-limited settings, manual removal is often combined with dipping and acaricides to manage livestock tick infestations [55]. Tick traps utilizing attractants like carbon dioxide effectively capture species such as *A. americanum*, *Hyalomma scupense*, and *I. scapularis*. These traps reduce tick populations and provide valuable data on tick behavior and population dynamics, aiding in targeted management strategies [288]. Host grooming, particularly for pets and livestock, physically removes ticks before attachment, reducing the likelihood of pathogen transmission and being effective against ticks like *R. microplus* [289].

### 5.2. Livestock Rotation and Restricting Host Access

A study by Cruz-González [290] highlights that livestock rotation mitigates tick infestations by managing grazing systems and reducing interactions with reservoir hosts, significantly lowering the prevalence of tick-borne diseases in livestock. Pasture rotation delays cattle contact with tick larvae, disrupting their life cycle. As a result, larvae die from starvation or dehydration [291]. Additionally, selective grazing can further help by using grasses with tick-repelling properties like *Mellinis minutiflora*, *Brachiaria brizantha*, and *Andropogon gayanus*, which produce natural repellents, preventing tick larvae from questing for their host [292]. Restricting wildlife access to livestock areas through fencing or barriers complements livestock rotation by limiting interactions between wildlife and livestock [293]

## 6. Molecular Control

The tick microbiota comprises diverse microbial populations, including pathogens, symbionts, and commensals, which collectively influence vectorial capacity, fitness, and pathogen transmission [294]. Research suggests that the tick microbiota can modulate pathogen establishment, development, and transmission by interacting with the host microbiota and influencing immune responses [295]. The ability of ticks to transmit pathogens such as *A. marginale*, *E. ruminantium*, and *C. burnetii* underscores the importance of studying the microbiota to identify novel targets for controlling tick infestations and associated diseases [296,297]. One promising strategy for tick control is paratransgenesis, which involves the genetic modification of tick-associated bacteria to express anti-pathogenic molecules that can interfere with pathogen development and transmission. Studies have shown that bacterial symbionts such as Coxiella-related organisms in *A. americanum* can impair the transmission of *E. chaffeensis*, indicating the potential to leverage symbiotic bacteria to block pathogen transmission [298]. Additionally, metagenomic approaches have identified various bacterial genera within ticks that can be genetically modified to disrupt vital biological processes such as digestion, ovogenesis, and vitellogenesis, thereby reducing tick reproduction and pathogen dissemination [299,300]. Paratransgenic strategies have been successfully applied to control other vectors like mosquitoes, and adapting these methods for ticks offers a viable, eco-friendly alternative to chemical acaricides, which are increasingly becoming ineffective due to resistance [301,302]. The tick microbiota not only affects vector competence but also plays a crucial role in tick development, nutrition, and immune defense. For example, symbiotic bacteria such as Francisella in *O. moubata* provide essential vitamins and nutrients necessary for tick survival, and their disruption results in compromised fitness and development [303]. Moreover, dysbiosis induced by pathogens such as *Theileria* sp. in *R. microplus* significantly alters the tick microbiome, leading to changes in vector competence and pathogen transmission dynamics [304]. Investigating these interactions can help identify microbial targets for developing anti-tick vaccines that alter the tick microbiome to impair pathogen transmission and reduce tick fitness [305]. Anti-tick microbiota vaccines that target specific bacterial populations have shown promise in laboratory settings, with the potential to disrupt pathogen colonization in the tick gut and salivary glands [306].

## 7. Vaccination

Recombinant protein vaccines, such as those based on Bm86 from the midgut of *R. microplus*, have shown significant efficacy by reducing tick infestations and decreasing the transmission of pathogens like *B. bovis* and *A. marginale* in cattle [307]. Other recombinant antigens, such as Subolesin-Major Surface Protein 1a, provide cross-protection against multiple tick species and pathogens, further broadening their applications [308]. Advancements in genetic vaccine technologies, including DNA- and mRNA-based vaccines, offer new pathways for TBD prevention. mRNA vaccines encoding multiple tick salivary proteins have shown the potential to impair tick feeding and reduce pathogen transmission, providing a rapid and adaptable method to address emerging TBDs [309]. Salivary proteins, such as Salp15 from *I. scapularis*, are critical vaccine targets due to their role in modulating host immune responses [310]. Vaccination against these proteins disrupts tick feeding and pathogen transmission, making them key components of next-generation vaccines [311,312,313]. Vaccines targeting specific tick proteins, such as Subolesin, have also demonstrated efficacy in reducing ectoparasite infestations and the prevalence of pathogens like *Babesia* and *Anaplasma* [314]. Similarly, inactivated vaccines against tick-borne encephalitis virus (TBEV) have provided robust protection in Europe and Asia, demonstrating the effectiveness of targeting specific pathogens [315].

### Challenges in Vaccination

Developing effective vaccines for tick control faces several challenges that must be addressed to ensure their success. One major challenge is the diversity of tick species, as they exhibit varied life cycles, host interactions, and blood-feeding patterns, making it difficult to create a universal vaccine [316]. Additionally, host diversity presents a significant hurdle, as vaccines need to protect multiple hosts, including humans, domestic animals, and wildlife, each with unique immune responses [68,317]. The genetic diversity of tick strains further complicates vaccine development, as variations in tick populations can impact vaccine efficacy and necessitate the inclusion of a broad range of antigenic targets [318]. Economic considerations also play a crucial role, as the large-scale implementation of vaccine strategies can be costly, limiting accessibility and widespread adoption, particularly in resource-limited regions [68]. The complexity of tick-borne diseases may require multi-antigen vaccines, incorporating antigens from various tick species or tick-borne pathogens to provide comprehensive protection [319]. Finally, the development of effective delivery platforms is essential to ensure efficient vaccine administration and optimal immune responses in the host [319].

## 8. Novel Strategies

Tick control strategies include various approaches such as vaccines, RNA interference (RNAi), genome editing, plant-derived acaricides, and integrated management strategies. DNA and mRNA vaccines have been studied for their potential to control tick infestations and pathogen transmission by targeting tick saliva proteins, such as Salp14, inducing tick resistance and preventing Lyme disease transmission [320,321]. Anti-tick microbiota vaccines target the tick microbiome to disrupt symbiotic relationships and reduce pathogen transmission, impacting the feeding performance of *I. ricinus* and reducing pathogen load in *I. scapularis* [305,322]. Cutaneous hypersensitivity vaccines are designed to trigger an immune response in the host that results in early tick detection and rejection, leading to reduced feeding efficiency in cattle [323]. RNAi technology is used to silence essential genes in ticks, affecting their survival and ability to transmit diseases, such as those related to reproduction and digestion, reducing tick viability [324]. The CRISPR/Cas9 system has been explored as a tool to modify tick genes and reduce their ability to transmit pathogens by targeting developmental genes in *I. scapularis*, leading to impaired molting and reproduction [325]. Plant-derived acaricides offer eco-friendly alternatives to synthetic chemicals, with essential oils from oregano and rosemary, as well as *Ximenia americana* stem bark extract, showing promising efficacy against *R. microplus* [160,326]. Integrated tick management strategies combine biological control, habitat management, and selective acaricide application to reduce tick populations sustainably, with the One Health approach recommended to address tick control holistically [327].

## 9. Integrated Control Approaches

### 9.1. The Role of Integrated Pest Management (IPM)

By integrating diverse methods, such as biological agents, cultural practices, and chemical treatments, IPM creates a resilient system capable of adapting to changing ecological conditions and pest dynamics [24]. Traditional tick control has largely relied on chemical acaricides; however, their extensive use has led to widespread resistance, environmental contamination, and residue accumulation in livestock products [158,328]. IPM offers a sustainable alternative by integrating various control methods, including chemical, biological, cultural, and mechanical interventions. Vaccination against ticks, specifically *R. microplus*, using Bm86-based vaccines such as TickGARD™ and Gavac™, has shown significant efficacy in reducing tick infestations and the incidence of tick-borne diseases. These vaccines, which induce an immune response that damages engorging ticks, serve as an effective complement to chemical control measures [329,330,331]. Additionally, biological control methods, such as entomopathogenic fungi like *M. anisopliae* and *B. bassiana*, show promise in managing tick populations with minimal impact on non-target organisms. These fungi infect ticks by penetrating their cuticle, leading to mortality, and are already commercially available for pest control [275,332]. Cultural practices such as rotational grazing and pasture spelling further contribute to breaking the tick life cycle and reducing infestation rates [158,290,328,333].

### 9.2. Success Stories in Integrated Tick Management

Several regions have successfully implemented integrated tick management programs, demonstrating the effectiveness of combining multiple strategies. In Cuba, the Bm86-based vaccine Gavac™, combined with strategic acaricide use, reduced *R. microplus* infestations by 87% over eight years. This approach significantly decreased babesiosis cases and mortality rates, improving cattle health and productivity while lowering acaricide dependence [334,335]. Similarly, in Australia, the introduction of TickGARD™ has been instrumental in reducing infestations of *R. microplus* and *R. annulatus*, thereby decreasing economic losses in the beef industry [336,337]. In Mexico, an integrated tick management (ITM) program in the tropical region of Veracruz successfully combined chemical acaricides with entomopathogenic fungi (*M. anisopliae*), plant extracts, pasture rotation, and Zebu cattle breeding, reducing tick infestations and acaricide reliance [338]. Mexico’s use of ferritin-based vaccines in combination with acaricide applications has shown promising results in controlling *R. annulatus* infestations [339]. The U.S. Cattle Fever Tick Eradication Program (CFTEP), initiated in the early 1900s, implemented systematic cattle dipping, quarantine measures, and surveillance, leading to the elimination of the CFT by 1943 [340]. In Brazil, the use of tick-repellent grasses such as *Melinis minutiflora* and *Andropogon gayanus*, which produce secondary metabolites with repellent properties, significantly reduced *R. microplus* larval populations in infested pastures [292].

### 9.3. Challenges in Implementing IPM

Despite its proven effectiveness, the implementation of IPM strategies faces several challenges. One of the primary obstacles is the high initial cost and knowledge requirement associated with adopting multiple control measures and training farmers to effectively implement them [158,341]. Additionally, resistance to change among farmers accustomed to traditional acaricide use poses a significant barrier to widespread adoption [342]. The lack of infrastructure for widespread vaccination programs and biological control measures further hinders the effective implementation of IPM in resource-limited settings [158,343]. Environmental factors, such as climatic variations and the availability of suitable pasture for rotational grazing, also influence the success of IPM programs [344,345]. Moreover, the development of resistance to vaccines and biological control agents, although slower compared to chemical acaricides, remains a concern that requires continuous research and the adaptation of control strategies [158,332].

#### Socio-Economic Constraints

Socio-economic factors significantly hinder effective tick control, particularly in resource-limited regions. High costs associated with acaricides, biological control agents, and habitat management often prevent small-scale farmers from implementing adequate measures, exacerbating infestations and their associated health impacts [55]. The global economic impacts of ticks and tick-borne diseases across various sectors are summarized in Table 6, illustrating the widespread financial burden on healthcare, agriculture, and public health.

## 10. Conclusions

Effective tick management requires an integrated approach that combines chemical, biological, and cultural strategies to address resistance and ensure environmental sustainability. Biological control methods, such as entomopathogenic fungi, parasitoids, natural repellents, and habitat management, offer eco-friendly alternatives that complement traditional chemical acaricides. Future efforts should focus on adapting strategies to regional conditions and advancing innovations like microbiota-targeted controls, next-generation vaccines, and nanotechnology-enhanced acaricides. Public education and stakeholder engagement are critical for widespread adoption and success. To reduce the impact of tick-borne diseases on public health and agriculture, scalable, cost-effective interventions grounded in One Health principles are essential. Collaborative, locally tailored approaches will pave the way for sustainable and effective tick management.

## 11. Patents

This study did not result in any patents.

## Figures and Tables

**Table 1:** Health impacts of ticks on humans and animals.

Aspect	Details	References
**Disease transmission**	Ticks are significant vectors of pathogens (bacteria, viruses, protozoa) causing diseases such as Lyme disease, anaplasmosis, babesiosis, and tick-borne encephalitis.	[9,10]
**Human health impact**	Range expansion increases zoonotic disease risks in non-endemic areas.	[11]
**Livestock health impact**	In livestock, ticks cause anemia, weight loss, reduced milk production, and mortality.	[12,13,14]
**Companion animal health**	In pets, ticks cause dermatitis and transmit diseases like ehrlichiosis, increasing the risk of disease transmission to humans.	[14]
**Economic impacts**	Tick infestations result in economic losses from reduced productivity, veterinary costs, and acaricide use.	[15,16]
**Climate-driven risks**	Climate change exacerbates tick abundance, geographic spread, and disease prevalence in humans and animals.	[3]

**Table 2:** Examples of tick species and their preferred hosts.

Tick Species	Preferred Hosts	References
*Amblyomma hebraeum*	Cattle, sheep, goats, large wild ruminants (e.g., giraffes, buffalo, eland), warthogs, and rhinoceroses	[87]
*Amblyomma variegatum*	Cattle, sheep, goats, and birds	[87]
*Hyalomma rufipes*	Cattle, sheep, goats, horses, large herbivores, birds, and scrub hares	[87]
*Rhipicephalus decoloratus*	Cattle, impalas, eland, nyalas, bushbuck, kudu, horses, and zebras	[87]
*R. microplus*	Domestic cattle, goats, grey rhebok, and eland	[87]
*Rhipicephalus appendiculatus*	Cattle, goats, African buffalo, eland, greater kudu, and sable antelope	[87]
*Haemaphysalis elliptica*	Dogs, cats, and rodents	[87]
*Rhipicephalus sanguineus*	Dogs, humans, and other mammals	[87]

**Table 3:** Classes of acaricides, their mechanisms of action, and examples. This table provides a detailed breakdown of commonly used acaricides, highlighting their modes of action and references to key studies.

Class of Acaricide	Examples	Mode of Action	References
Organophosphates	Diazinon and chlorpyrifos	Inhibits acetylcholinesterase, leading to acetylcholine accumulation, paralysis, and death.	[23,155,156,157]
Carbamates	Carbaryl	Inhibits acetylcholinesterase similar to organophosphates but with shorter residual effects.	[23,155,156,157]
Pyrethroids	Permethrin, cypermethrin, and deltamethrin	Acts on sodium channels, causing hyperexcitation, paralysis, and death.	[23,155,156,157]
Macrocyclic lactones	Ivermectin and moxidectin	Targets glutamate-gated chloride channels, leading to paralysis and death.	[23,155,156,157]
Insect Growth Regulators (IGRs)	Methoprene and diflubenzuron	Disrupts molting and reproduction by mimicking hormones.	[23,155,156,157]
Phenylpyrazoles	Fipronil	Blocks GABA receptors, causing uncontrolled neural activity and death.	[23,155,156,157]
Chitin synthesis inhibitors	Etoxazole and novaluron	Prevents exoskeleton formation, disrupting growth and molting.	[158,159]
Biological acaricides	Neem extract and clove extract	Multiple mechanisms including neurotoxic and metabolic disruption.	[160]

**Table 4:** Key natural tick repellents: botanical sources, active compounds, and their efficacy against tick species.

Natural Repellent	Source	Active Compounds	Efficacy	References
Catnip oil	*Nepeta cataria*	Nepetalactone	84% repellent effect after 2 h	[198]
Eucalyptus oil	*Eucalyptus globulus*	Eucalyptol	97% acaricidal mortality at 10% concentration	[199]
Garlic extract	*Allium sativum*	Allicin	Significant repellent activity: 87% (males) and 87.5% (females); 4% avoidance	[187]
Cinnamon oil	*Cinnamomum verum*	Cinnamaldehyde	68–97% (*Hae. longicornis*), 69–94% (*R. haemaphysaloides*), and 69–93% (*H. asiaticum*)	[200]
Tobacco extract	*N. tabacum*	Nicotine and anatabine	100% mortality at 36 h (60% concentration) and 100% mortality at 48 h (all concentrations) in *R. microplus*	[201]
Rosemary oil	*Rosmarinus officinalis*	Carnosic acid	Effective against *I. ricinus*	[202,203,204,205,206]
Clove oil	*Syzygium aromaticum*	Eugenol	Proved to be effective against *R. microplus* larvae and adult ticks	[207]
Lippia alba oil	*Lippia alba*	Citral	Effective against *R. microplus*	[208]
Geranium oil	*Pelargonium* spp.	Geraniol	Effective against *I. ricinus*	[209]
Nootkatone	Alaskan cedar tree	Nootkatone	Effective against *I. scapularis*	[197]

**Table 5:** Overview of tick acaricide resistance by region, species, and chemical class. This table highlights the geographic distribution of acaricide-resistant tick species.

Country	Tick Species	Chemical Class Resistance	References
Cameroon	*R. microplus*	Organophosphates, synthetic pyrethroids, amidines, and macrocyclic lactones	[223,224]
Zambia	*R. decoloratus*, *Rhipicephalus* spp., and *Am. variegatum*	Organophosphates and synthetic pyrethroids	[225]
Brazil	*R. microplus*	Organophosphates, synthetic pyrethroids, formamidines, and macrocyclic lactones	
South Africa	*R. microplus*	Organophosphates	[226]
Mexico	*R. microplus*	Organophosphates and synthetic pyrethroids	[227]
Côte d’Ivoire	*R. microplus*	Alpha-cypermethrin, deltamethrin, and amitraz	[228]
Uganda	*R. decoloratus* and *Rhipicephalus* spp.	Organophosphates and synthetic pyrethroids	[229]
Colombia	*R. microplus*	Pyrethroids and organophosphates	[230,231]
Burkina Faso	*R. microplus* and *A. variegatum*	Cypermethrin and deltamethrin	[232]
India	*R. microplus*	Organophosphates and synthetic pyrethroids	[233]
Pakistan	*Hy. anatolicum*	Organophosphates and synthetic pyrethroids	[234]
United Arab Emirates	*H. dromedarii*	Organophosphates and synthetic pyrethroids	[235,236]
Argentina	*R. microplus*	Ivermectin and organophosphates	[237,238]
Cameroon	*R. microplus*	Organophosphates, synthetic pyrethroids, amidines, and macrocyclic lactones	[239]

**Table 6:** Summary of economic impacts of ticks and tick-borne diseases worldwide (costs converted to USD).

Country	Estimated Annual Cost (USD)	References
United States	USD 345–968 million	[46]
Holland	USD 20 million	[346]
India	USD 498.7 million	
Brazil	USD 32.4 million	[347]
South Africa	USD 68.6 million	[348]
Germany	USD 40 million	[346]
Kenya	USD 7.0 million	[349]
Mexico	USD 573.16 million	[350]
Colombia	USD 168.0 million	[350]
Australia	USD 250.0 million	[53]
Tanzania	USD 364.0 million	[53]
Puerto Rico	USD 6.7 million	[351]
Zambia	USD 5.0 million	[351]

## Data Availability

No new data were created or analyzed in this study.
